# Low-dose YC-1 combined with glucose and insulin selectively induces apoptosis in hypoxic gastric carcinoma cells by inhibiting anaerobic glycolysis

**DOI:** 10.1038/s41598-017-12929-9

**Published:** 2017-10-04

**Authors:** Kota Wakiyama, Yoshihiko Kitajima, Tomokazu Tanaka, Masao Kaneki, Kazuyoshi Yanagihara, Shinichi Aishima, Jun Nakamura, Hirokazu Noshiro

**Affiliations:** 1 Department of Surgery, Saga University Faculty of Medicine, 5-1-1 Nabeshima, Saga, 849-8501 Japan; 2 Department of Surgery, NHO Higashisaga Hospital,7324 Harakoga, Miyaki-cho, Miyaki-Gun, Saga, 849-0101 Japan; 3 Department of Anesthesia, Critical Care and Pain Medicine, Massachusetts General Hospital, Harvard Medical School, Boston, USA; 4 Division of Biomarker Discovery, Exploratory Oncology Research & Clinical Trial Center, National Cancer Center, 6-5-1 Kashiwanoha, Kashiwa, Chiba, 277-8577 Japan; 5 Department of Pathology, Saga University Faculty of Medicine, 5-1-1 Nabeshima, Saga, 849-8501 Japan

## Abstract

This study aimed to establish a therapeutic strategy targeting hypoxic cancer cells in gastric carcinoma (GC). YC-1 is a HIF-1α inhibitor, and we revealed that low-dose YC-1 (10 µM) suppressed HIF-1α expression, and induced hypoxia-dependent apoptosis in the GC cell line 58As9. This hypoxia-specific apoptosis induction by YC-1 involved excessive reactive oxygen species (ROS) generation. The apoptotic effect of 10 µM YC-1 was enhanced by additional glucose (G) and insulin (I) treatments. RT-PCR demonstrated that 10 µM YC-1 reduced hypoxia-induced expression of HIF-1α targets involved in anaerobic glycolysis. Metabolic analysis showed that YC-1 shifted glucose metabolism in hypoxic cells from anaerobic glycolysis to oxidative phosphorylation (OXPHOS). Additional GI accelerated membranous GLUT1 translocation, elevating glucose uptake, and increased acetyl-CoA levels, leading to more ROS generation in hypoxic YC-1-treated cells. Finally, we evaluated the anti-cancer effect of low-dose YC-1 (1 mg/kg) + G (2 g/kg) and I (1 unit/3 g G) treatment in xenograft models. YC-1 + GI therapy strongly inhibited tumour growth. Immunohistochemical analysis demonstrated that YC-1 + GI reduced HIF-1α expression and pimonidazole accumulation in tumours. Conversely, YC-1 + GI increased intra-tumoral 8-OHdG and levels of apoptosis markers. Low-dose YC-1 + GI is a unique therapy targeting hypoxic GC cells that generates lethal ROS via forced activation of OXPHOS.

## Introduction

Intratumoral hypoxia (low O_2_) is a common characteristic of many solid tumours^1,2^. HIF-α (HIF-1α or HIF-2α), a basic-helix-loop-helix transcription factor, functions as a master regulator of oxygen homeostasis. Under normoxia, prolyl hydroxylases (PHDs) use oxygen as a substrate to hydroxylate key proline residues within HIF-α, which is then degraded through the proteasomal pathway following pVHL-mediated ubiquitination. Under hypoxia, PHD activity is inhibited, and HIF-α is stabilized, forming an active complex with aryl hydrocarbon receptor nuclear translocator (ARNT), and upregulates hundreds of target genes through binding hypoxia-response elements (HREs)^3–5^. HIF-α overexpression has been found in many human cancers and is associated with the induction of genes implicated in angiogenesis, tumour metabolism, invasion, metastasis and radio- and chemo-resistance^6–11^, which contribute to poor patient survival^11^. Therefore, inhibition of HIF-α is an attractive strategy for cancer therapy; however, no selective HIF-α inhibitor has been clinically approved^12–15^.

Recently, we reported that HIF-1α knockdown (KD) by siRNA induces apoptosis in the gastric carcinoma (GC) cell line 58As9 under hypoxia. This hypoxia-dependent apoptosis was induced by excessive production of reactive oxygen species (ROS), whereby HIF-1α KD inhibited hypoxic induction of genes involved in the ROS control system including anaerobic glycolysis in 58As9 cells^16^. This study further revealed that hypoxia-induced apoptosis was accelerated by additional glucose (G) and insulin (I) treatments in the KD cells, as higher ROS generated via increased glucose uptake^16^. Based on this study, we attempted to establish a novel anti-cancer therapy using a specific HIF-1α inhibitor combined with GI to target hypoxic cancer cells in gastric tumours.

ROS are mainly generated in the mitochondria by oxidative phosphorylation (OXPHOS), a process performed by the electron transport chain (ETC)^17–21^. Excessive ROS generation is known to cause ROS-mediated damage to nucleic acids, proteins and lipids, leading to cell death^18–21^. It has been reported that ROS are increased in hypoxic cancer cells, and HIF-1α induction plays an adaptive mechanism in controlling ROS generation via up-regulating genes involved in anaerobic glycolysis^3,15,16,19^. In the anaerobic glycolysis pathway, HIF-1α first activates GLUT1 transcription to increase glucose uptake in cells^22^. Glucose is then metabolized to pyruvate by the actions of glycolytic members including ALDOC^23^. Under aerobic conditions, pyruvate is converted to acetyl-CoA by pyruvate dehydrogenase (PDH) for entry into the tricarboxylic acid (TCA) cycle^18^. Conversely, in cancer cells exposed to hypoxia, pyruvate is shunted away from the mitochondria by HIF-1α-mediated PDK1 upregulation, which inhibits PDH activity. Thereafter, LDHA alternatively converts pyruvate to lactate and MCT4 effluxes the lactate^24–26^. Together, these reports indicate that HIF-1α is a central molecule in suppressing excessive ROS production in hypoxic cells via up-regulating target genes involved in anaerobic glycolysis.

YC-1 [3-(5′-hydroxymethyl-2′-furyl)-1-benzylindazole] was originally developed as a potential therapeutic agent for circulation disorders because of its inhibitory effect on platelet aggregation and vascular contraction^27^. Recent studies have found that YC-1 blocked HIF-1α expression at the post-transcriptional level and consequently inhibits the transcription factor activity of HIF-1 in cancer cells under hypoxia^28–30^. However, no study has assessed the anti-cancer effect of YC-1 on cancer metabolism under hypoxia.

In this study, we first determined the optimal dose of YC-1 that effectively inhibited HIF-1α expression and induced hypoxia-dependent apoptosis in GC cells. We next analyzed whether additional GI treatment enhanced this apoptotic effect. Metabolic analysis addressed the mechanism of YC-1 + GI-induced apoptosis in cells under hypoxia. Finally, we assessed whether this combination therapy selectively induced apoptosis in hypoxic cancer cells *in vivo*.

## Results

### Growth inhibition by YC-1 treatment in GC cells

**Figure 1:**
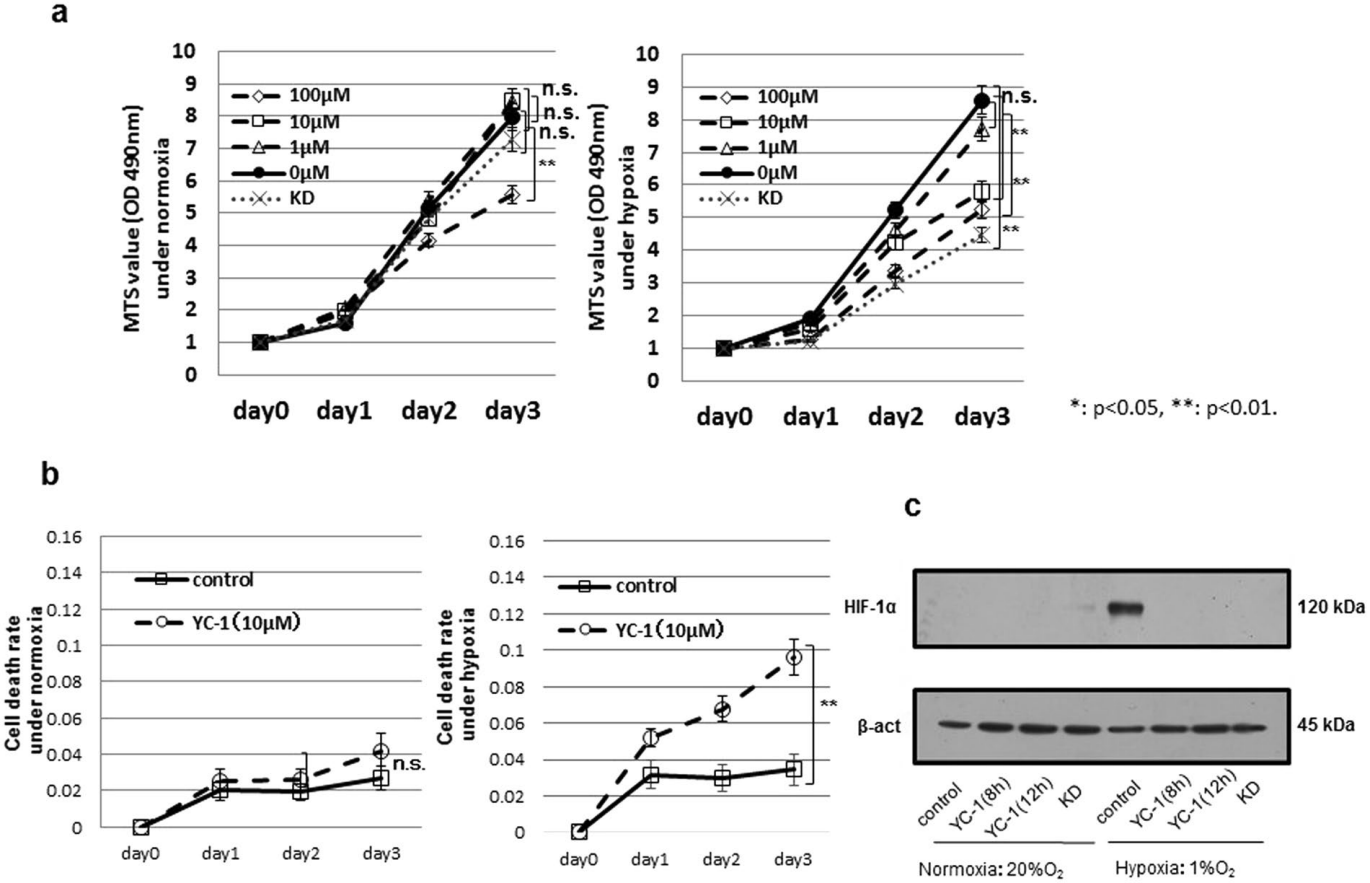
YC-1 inhibited growth in the gastric cancer cell line 58As9. (**a**) Viability of YC-1-treated 58As9 cells under normoxia and hypoxia; viability of HIF-1α KD (KD) cells was evaluated in parallel. (**b**) Cell death rate in 58As9 cells with or without YC-1 (10 μM) under normoxia and hypoxia. (**c**) Western blot analysis of HIF-1α expression in 58As9 cells under normoxia and hypoxia with or without YC-1 (10 μM) treatment for 8 and 12 h. β-actin was equally expressed in all cells. ns: not significant, **p < 0.01.

The GC cell line 58As9 was treated with YC-1 at 1, 10 and 100 μM, and cell viability was evaluated by the MTS assay (Fig. 1a). The results showed that 1 μM YC-1 did not influence cell viability, while 10 μM YC-1-treated 58As9 and KD cells showed significantly decreased viability under hypoxia but not normoxia. At 100 μM, YC-1 decreased viability under both normoxia and hypoxia (Fig. 1a). YC-1 exhibited similar effects in another GC cell line, MKN74 (Supplemental Fig. 1). Cell death was next evaluated in 58As9 cells with or without 10 μM YC-1 (Fig. 1b). Under normoxia, there was no difference in the cell death rate between controls (no YC-1) and 10 μM YC-1. The cell death rate significantly increased under hypoxia with 10 μM YC-1, but not in controls (Fig. 1b). WB analysis showed that HIF-1α was elevated by hypoxia in control cells, while the hypoxic induction of HIF-1α was inhibited in YC-1-treated 58As9 and KD cells (Fig. 1c).

### YC-1 plus GI treatment induced apoptosis in 58As9 cells under hypoxia

**Figure 2:**
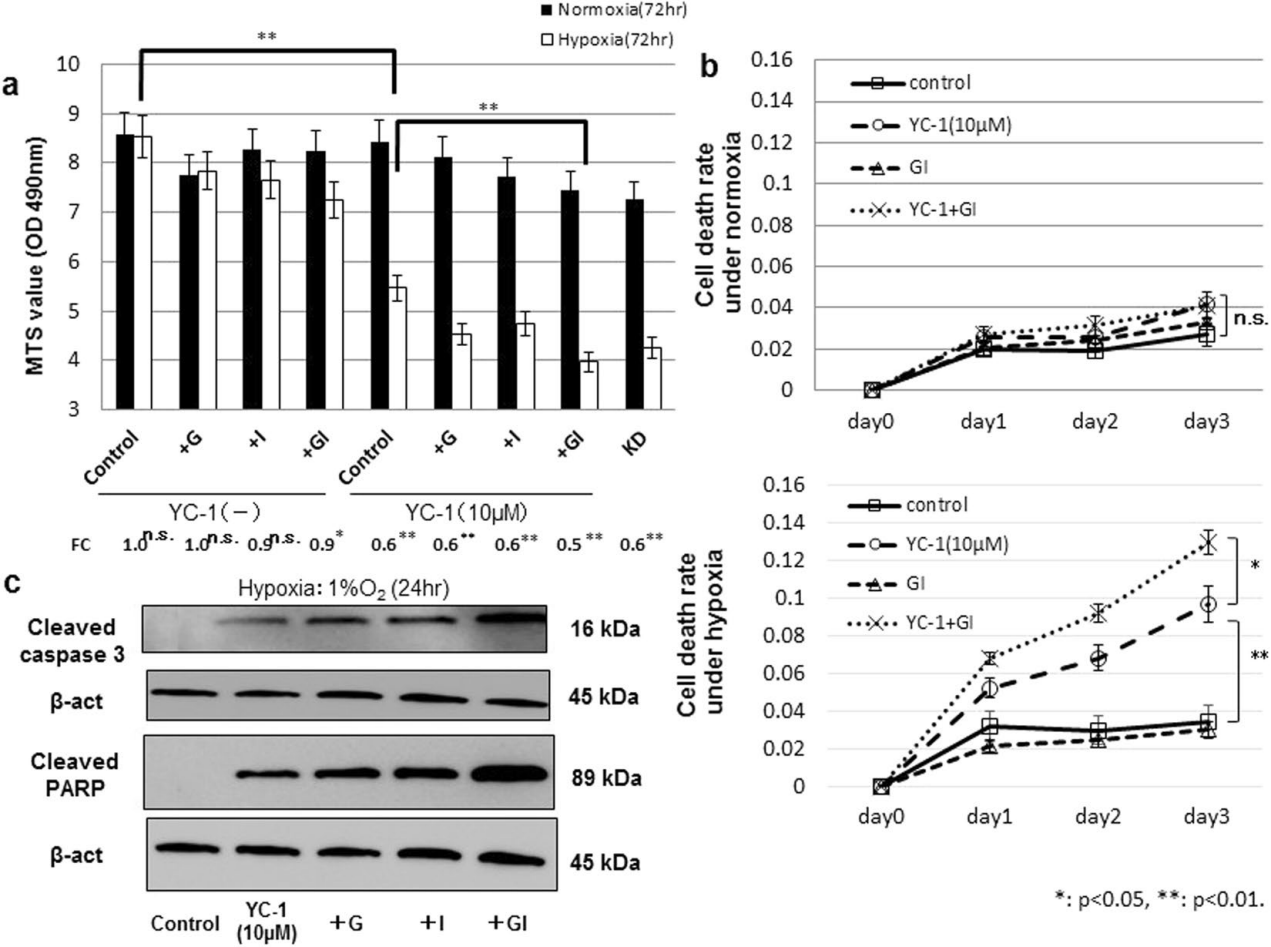
Hypoxia-dependent apoptosis in 58As9 cells with YC-1 or YC-1 + GI treatment. (**a**) Cell viability in control and 10 μM YC-1-treated cells under normoxia or hypoxia was analyzed in the presence of G and/or I for 72 h. Fold change (FC) of hypoxia/normoxia is presented at the bottom. (**b**) Cell death rate in YC-1-, GI- and YC-1 + GI-treated cells under normoxia or hypoxia for 3 d. (**c**) Western blot analysis of cleaved-caspase3 and cleaved-PARP in YC-1-, YC-1 + G-, YC-1 + I- and YC-1 + GI-treated cells under hypoxia for 24 h. β-actin was equally expressed in all cells. ns: not significant, *p < 0.05, **p < 0.01.

We next evaluated the effect of additional GI treatment on hypoxia-dependent cell death in 10 μM YC-1-treated 58As9 cells. Cell viability was evaluated in control (no YC-1) and 10 μM YC-1-treated cells in the presence of glucose (G) and/or insulin (I) (Fig. 2a). In the control group, cell viability was unchanged between normoxia and hypoxia in control, G and I treatments, whereas viability decreased under hypoxia in GI treatment (fold change (FC) of hypoxia/normoxia: 0.9) (Fig. 2a). In the 10 μM YC-1 group, cell viability was more strongly inhibited under hypoxia than normoxia in all treatments (Fig. 2a). The lowest FC was found in the YC-1 + GI (FC: 0.5) treatment. Further, in the YC-1 group, cell viability under hypoxia was significantly lower in GI treatment than controls (Fig. 2a). The strong inhibitory effect of YC-1 + GI on cell viability was also observed in hypoxic MKN74 cells (Supplement Fig. 2). As shown in Fig. 2b, the cell death rate was not different among control, 10 μM YC-1, GI and YC-1 + GI under normoxia. However, the cell death rate was significantly increased in 10 μM YC-1 and YC-1 + GI compared with controls, and there was a higher death rate in YC-1 + GI than 10 μM YC-1 alone (Fig. 2b). WB analysis was used to evaluate apoptotic cell death in hypoxic 58As9 cells treated with YC-1 and/or GI (Fig. 2c). Expression of the apoptosis markers cleaved-caspase3 and cleaved-PARP was increased in hypoxic 58As9 cells by the four treatments, but not controls (Fig. 2c). The highest expression of apoptosis markers was observed in YC-1 + GI-treated cells (Fig. 2c).

### Assessing ROS production after YC-1 + GI treatment under hypoxia

**Figure 3:**
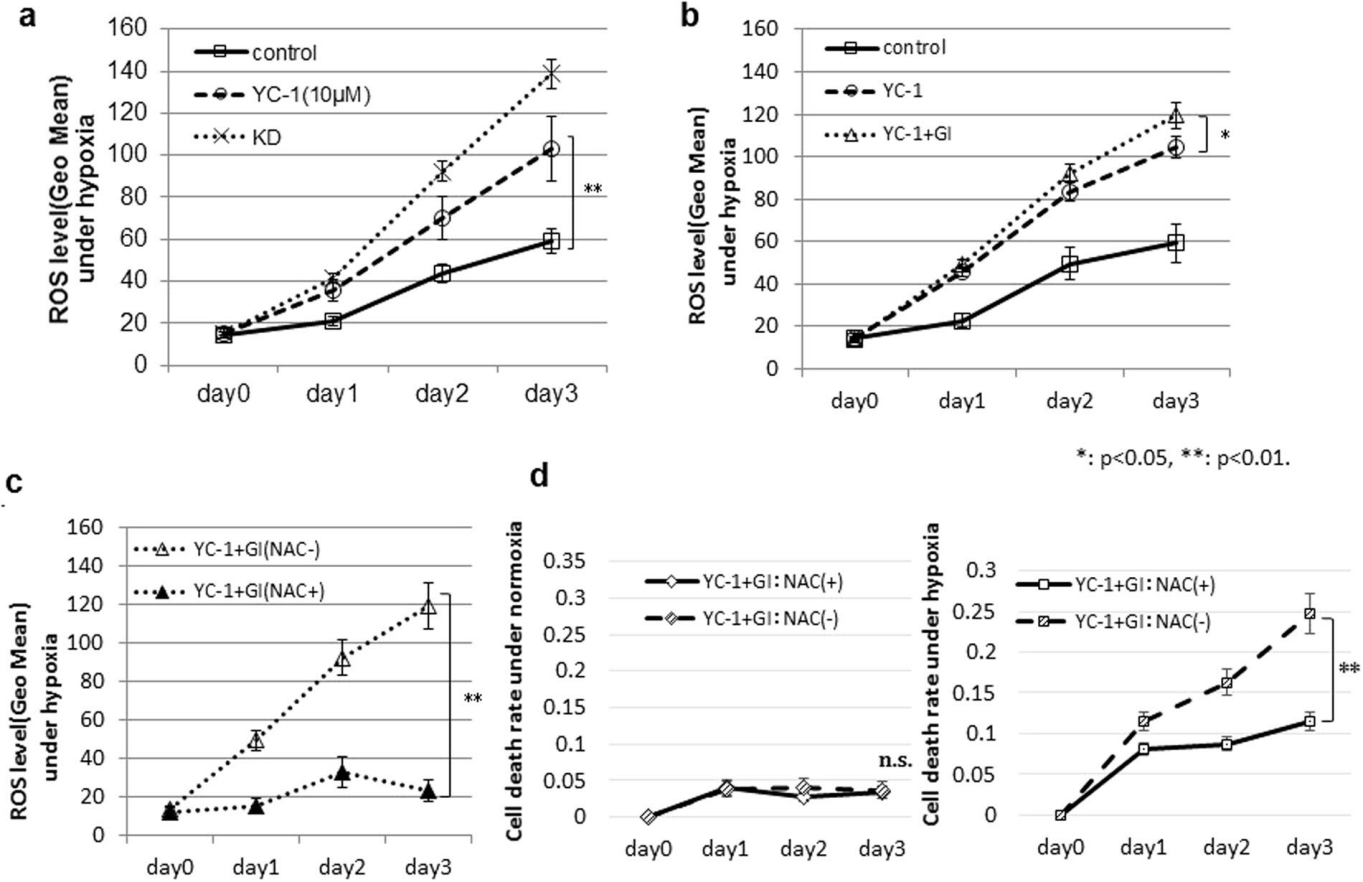
Effect of YC-1 + GI on ROS generation under hypoxia. (**a**) ROS levels in 58As9 cells with or without 10 μM YC-1 and in KD cells were analyzed under hypoxia for 3 d. (**b**) ROS levels in YC-1- or YC-1 + GI-treated cells under hypoxia. (**c**) ROS levels in YC-1 + GI-treated cells with or without 5 μM NAC. (**d**) Cell death rate in YC-1 + GI-treated cells with or without NAC under normoxia and hypoxia. ns: not significant, *p < 0.05, **p < 0.01.

We analyzed ROS levels in 58As9 cells under hypoxia with or without 10 μM YC-1 (Fig. 3a). Compared with controls (no YC-1), 10 μM YC-1 more strongly elevated ROS production in a time-dependent manner in hypoxic 58As9 cells (Fig. 3a). ROS levels on day 3 were significantly higher in 10 μM YC-1-treated cells than control cells under hypoxia, while the highest ROS was produced in hypoxic KD cells. ROS levels in hypoxic 58As9 cells were significantly higher in YC-1 + GI than in YC-1 on day 3 (Fig. 3b). Moreover, elevated ROS was blocked by the antioxidant NAC in YC-1 + GI-treated cells under hypoxia (Fig. 3c). The cell death rate was assessed with or without NAC in YC-1 + GI-treated cells (Fig. 3d). Under normoxia, the cell death rate was not different between the NAC (−) and NAC (+). In contrast, the cell death rate was significantly higher in the NAC (−) than NAC (+) under hypoxia on day 3 (Fig. 3d).

### RT-qPCR analysis of genes involved in anaerobic glycolysis

**Figure 4:**
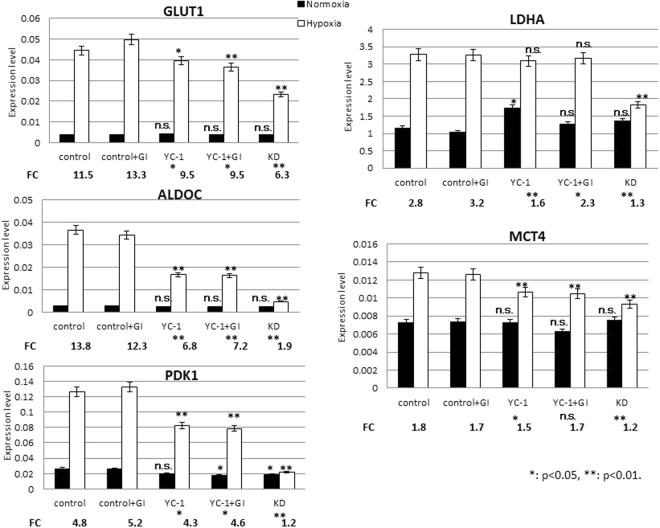
Expression of HIF-1α targets essential to anaerobic glycolysis. RT-qPCR analysis of five genes in control and GI-, YC-1- and YC-1 + GI-treated cells under normoxia and hypoxia; the expression levels are presented together with those in KD cells. FC of hypoxia/normoxia is presented at the bottom of each graph. mRNA expression levels under hypoxia were compared between control and treatment groups for five genes. ns: not significant, *p < 0.05, **p < 0.01.

RT-qPCR was used to evaluate hypoxia-induced expression of HIF-1α target genes in 58As9 cells in the control, GI, 10 μM YC-1 and YC-1 + GI groups (Fig. 4). The FC of hypoxia/normoxia was significantly decreased compared with controls by YC-1 and YC-1 + GI for all genes, except MCT4 in YC-1 + GI. However, KD cells showed the lowest FC for all five genes (Fig. 4). Levels of GLUT1, ALDOC, PDK1 and MCT4 expression under hypoxia were significantly decreased in YC-1 and YC-1 + GI to similar degrees, compared with controls. Expression of all five genes was most strongly suppressed in hypoxic KD cells (Fig. 4).

### Glucose uptake analysis

**Figure 5:**
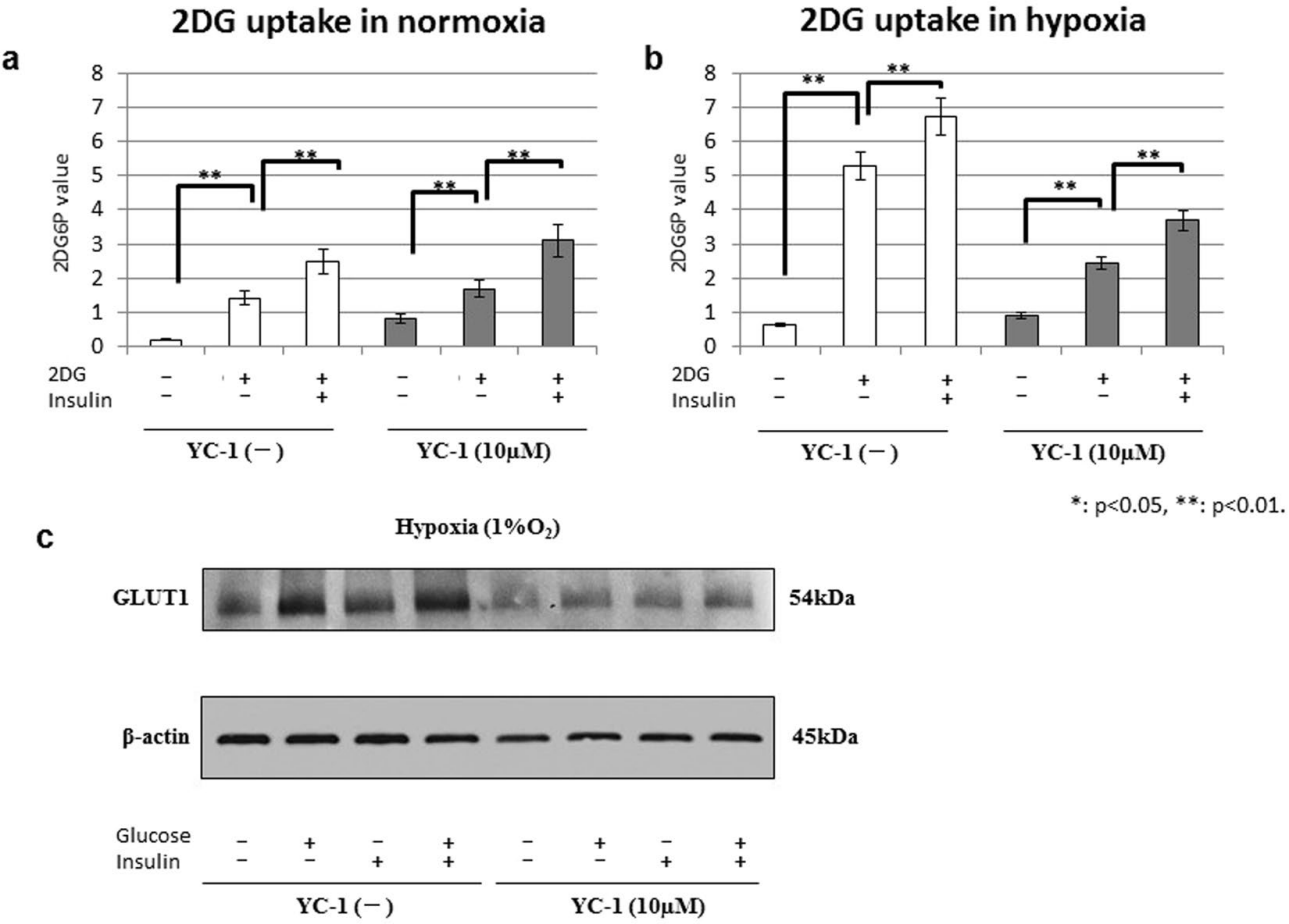
Glucose uptake in 58As9 cells with or without YC-1 + GI. (**a**,**b**) 2DG uptake levels in control and YC-1-treated cells were analyzed under normoxia (**a**) and hypoxia (**b**) for 24 h in the presence or absence of insulin. (**c**) Western blot analysis of membranous GLUT1 (54 kDa) levels in control and YC-1-treated cells that were cultured under hypoxia for 24 h in the presence or absence of G and/or I. β-actin was equally expressed in all cells. ns: not significant, **p < 0.01.

To assess glucose uptake, the 2DG uptake test was performed in 58As9 cells (Fig. 5a–b). As shown in Fig. 5a, 2DG uptake was elevated by insulin in 58As9 cells with and without YC-1 under normoxia. Under hypoxia, 2DG uptake was strongly increased in control (no YC-1) cells, and additional insulin treatment further elevated uptake (Fig. 5b). In 10 μM YC-1-treated cells, 2DG uptake was also promoted by insulin, but to a lower degree than in control cells (Fig. 5b). We next analyzed membranous GLUT1 expression in control and 10 μM YC-1 cells under hypoxia, in combination with G and/or I treatments (Fig. 5c). In the control group, membranous GLUT1 was increased with G or I, and most strongly elevated by GI. In the 10 μM YC-1 group, membranous GLUT1 was entirely reduced, compared with controls (Fig. 5c). Among treatments, the highest GLUT1 expression was observed in GI (Fig. 5c).

### Alteration in glucose metabolism by YC-1 + GI treatment

**Figure 6:**
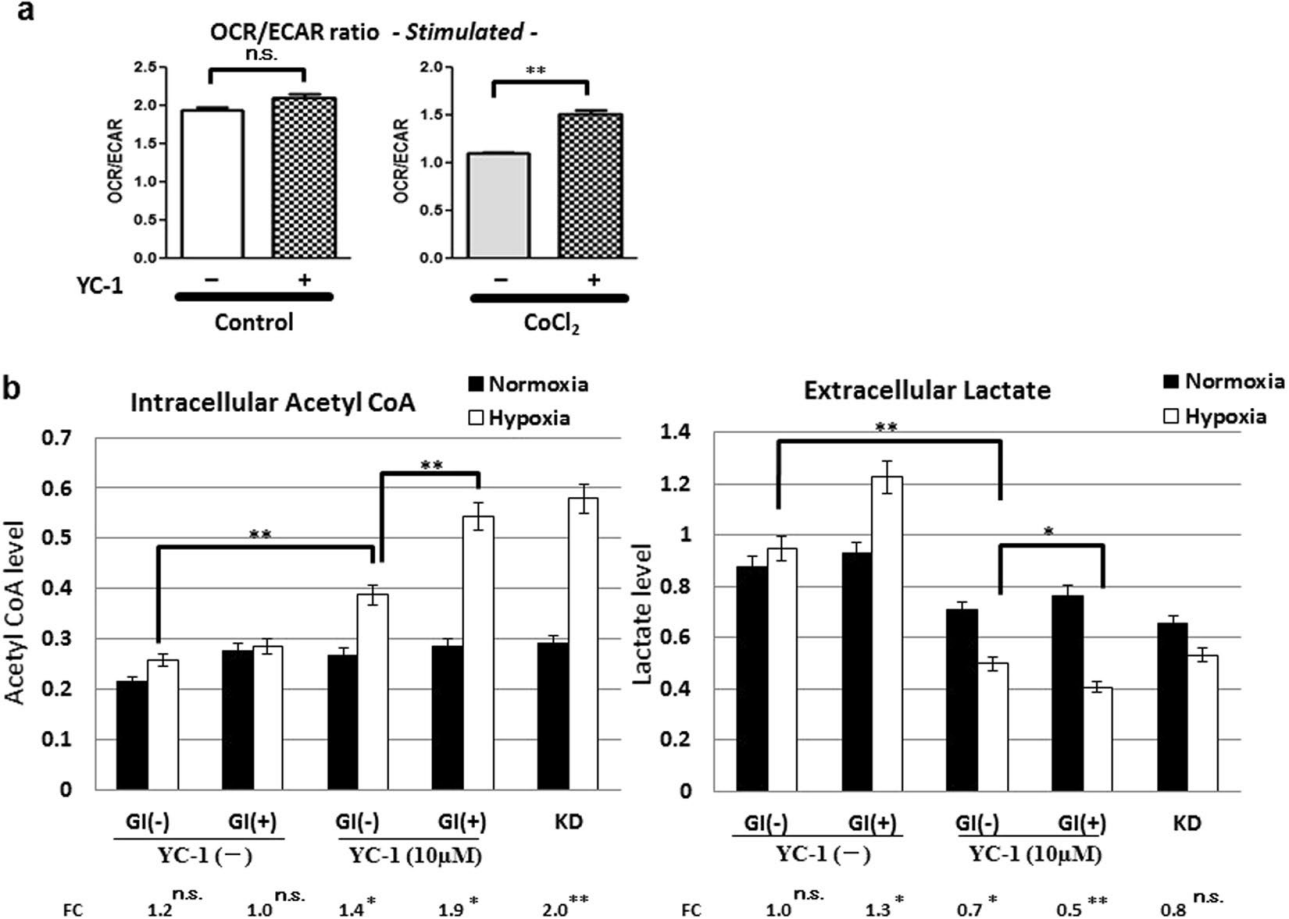
Reprograming glucose metabolism by YC-1 or YC-1 + GI treatment. (**a**) Using a Seahorse XF extracellular flux analyzer, OCR and ECAR were measured with or without the hypoxia-mimetic CoCl2. (**b**) Intracellular acetyl-CoA and extracellular lactate levels were measured in control, 10 μM YC-1 or KD cells under normoxia and 24 h hypoxia. In control and YC-1-treated cells, metabolite levels were further analyzed with or without GI treatment. FC values of hypoxia/normoxia are presented on the bottom of each graph. Acetyl-CoA and lactate levels under hypoxia were statistically compared as indicated in the graphs. ns: not significant, *p < 0.05, **p < 0.01.

OCR and ECAR were measured in absence or presence of the hypoxia-mimetic cobalt chloride (CoCl_2_) (Fig. 6a). The OCR/ECAR ratio was unchanged between YC-1 (−) and YC-1 (+) cells in the absence of CoCl_2_. In contrast, the OCR/ECAR ratio was significantly higher in YC-1 (+) than YC-1 (−) cells in the presence of CoCl_2_ (Fig. 6a).

We further measured glucose metabolites in YC-1 (−) and YC-1 (+) cells with or without GI (Fig. 6b). In the YC-1 (−) group, there was no significant difference in intracellular acetyl-CoA between normoxia and hypoxia (Fig. 6b). However, in the 10 μM YC-1 group, intracellular acetyl-CoA was significantly elevated under hypoxia in GI (−) cells with an FC of 1.4, and this hypoxic induction was further increased in GI (+) cells with an FC of 1.9 (Fig. 6b). In the 10 μM YC-1 group under hypoxia, acetyl-CoA was significantly higher in GI (+) than GI (−) (Fig. 6b). Extracellular lactate was next assessed (Fig. 6b); in the YC-1 (−) group, lactate levels were increased by hypoxia in the GI (+) (FC: 1.3) (Fig. 6b). In the 10 μM YC-1 group, lactate levels were significantly decreased under hypoxia compared with normoxia in both the GI (−) (FC: 0.7) and GI ( + ) (FC: 0.5). Furthermore, in the 10 μM YC-1 group under hypoxia, extracellular lactate was significantly lower in GI (+) than GI (−) (Fig. 6b).

### YC-1 plus GI treatment suppressed xenograft tumour growth in nude mice

**Figure 7:**
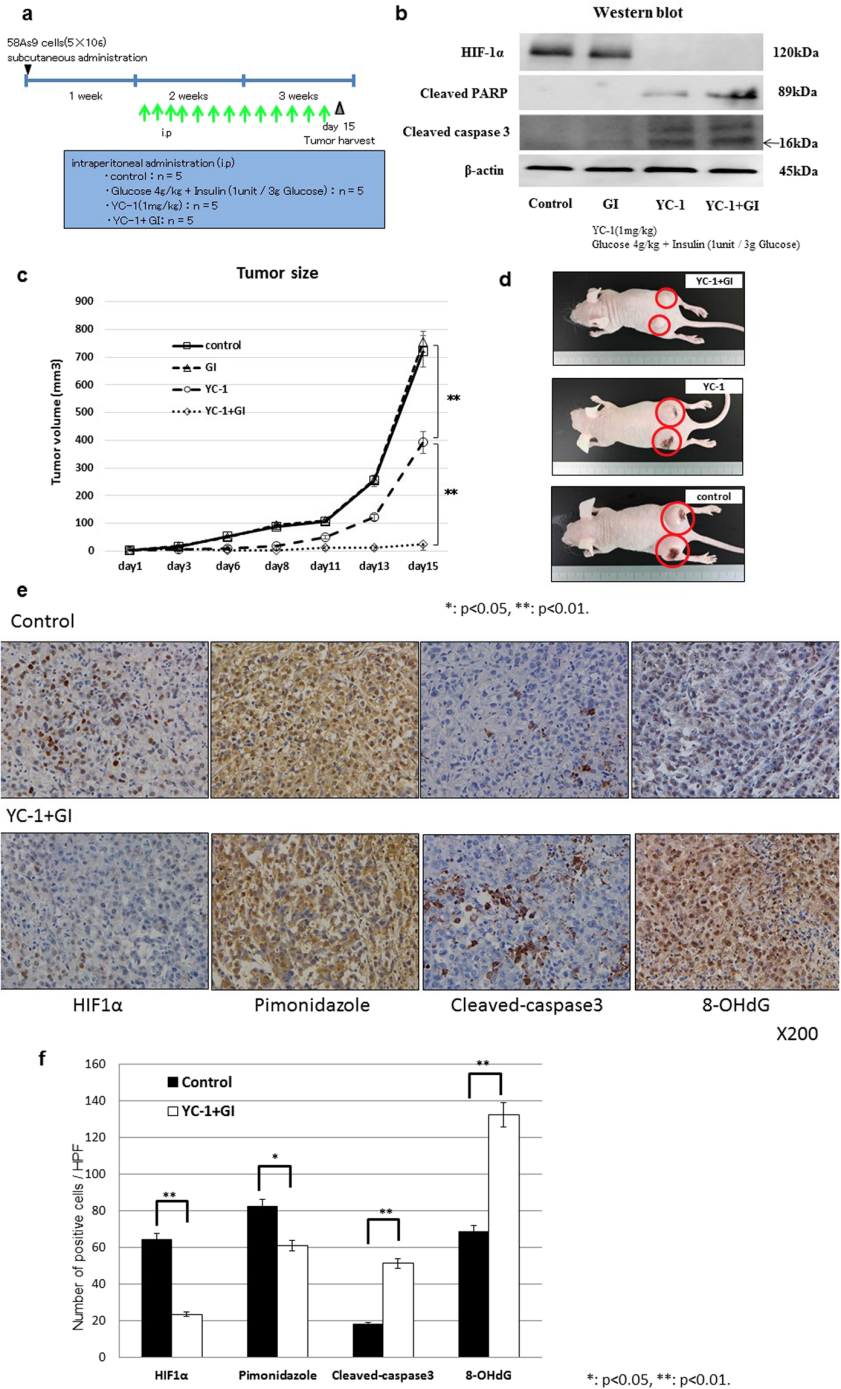
*In vivo* effect of YC-1 + GI treatment on tumour xenografts. (**a**) Experimental schedule of the YC-1 + GI treatment. The day 58As9 cells were subcutaneously injected is indicated by a triangle. The daily ip injections are shown by green arrows. Tumours were harvested on day 15, which is indicated by a reticulated triangle. Doses of drug treatments including control, glucose, YC-1 and YC-1 + GI are shown in the blue square. (**b**) Western blot analysis of HIF-1α, cleaved-caspase3 and cleaved-PARP in tumour xenografts from control, GI, YC-1 and YC-1 + GI groups. (**c**) Tumours sizes in the control, GI, YC-1 and YC-1 + GI groups were estimated on the indicated days. Mean value ± SE of tumor volumes was calculated and plotted on the graph. (**d**) Pictures of tumours treated with YC-1 + GI, YC-1 or control. (**e**) Immunohistochemical analysis of HIF-1α, pimonidazole, cleaved-caspase3 and 8-OHdG expressions in xenograft tumours from control or YC-1 + GI mice. (**f**) Comparison of the number of positively-stained cells from HIF-1α, pimonidazole, cleaved-caspase3 and 8-OHdG immunohistochemistry between control and YC-1 + GI treatment. ns: not significant, *p < 0.05, **p < 0.01.

Finally, we evaluated the *in vivo* effect of YC-1 + GI treatment in tumour xenografts (Fig. 7). The four drugs were ip injected into mice from day 1 to day 14, as shown in Fig. 7a. On day 15, tumours were harvested and subjected to WB analysis. HIF-1α expression was observed in control and GI mice, while its expression was inhibited in YC-1 and YC-1 + GI (Fig. 7b). In contrast, cleaved-PARP and cleaved-caspase3 were present in YC-1 and YC-1 + GI, and the levels were higher in YC-1 + GI than YC-1 (Fig. 7b). Figure 7c shows the growth curves of xenograft tumours that underwent the four treatments. There was no significant difference in size between control and GI tumors on day 15 (Fig. 7c). In contrast, tumour sizes of YC-1 or YC-1 + GI were significantly smaller than control, and tumour growth in YC-1 + GI was more strongly inhibited than in YC-1 (Fig. 7c). In the representative images of tumor-bearing mice, tumours appeared to be smaller in order of control, YC-1 and YC-1 + GI (Fig. 7d). In this model, no mice died in any treatment. Immunohistochemistry evaluated levels of HIF-1α, pimonidazole, cleaved-caspase3 and an oxidized base, 8-OHdG, in xenograft tumours that underwent control or YC-1 + GI treatments (Fig. 7e). HIF-1α and pimonidazole staining appeared to be stronger in control than YC-1 + GI-treated tumours, whereas cleaved-caspase3 and 8-OHdG staining were stronger in YC-1 + GI (Fig. 7e). Statistical analysis demonstrated that the number of positive HIF-1α and pimonidazole cells was significantly higher in control than YC-1 + GI, while cleaved-caspase3 and 8-OHdG were higher in YC-1 + GI (Fig. 7f).

## Discussion

In this study, we first attempted to isolate a compound that inhibited cell viability in 58As9 cells specifically under hypoxia, similar to what we had previously shown for HIF-1α KD 58As9 cells^16^. Many small molecules have been reported to be HIF-1α inhibitors^12–15^. We explored a desirable drug that reduced cell viability specifically in hypoxia, but not normoxia among the 15 known HIF-1α inhibitors^12–15^. The MTS assay determined that only 10 μM YC-1 decreased cell viability in 58As9 cells selectively in hypoxia and inhibited HIF-1α expression. These results suggest low-dose YC-1 (10 μM) is essential for hypoxia-dependent cell growth inhibition. Conversely, high-dose YC-1 (100 μM) decreased cell viability under both normoxia and hypoxia. The inhibitory effect of 100 μM YC-1 on cell viability under normoxia may be derived from unknown HIF-1α-independent mechanisms. In this study, we did not assess whether YC-1 treatment inhibits HIF-2α expression, or whether HIF-2α KD affects cell growth in hypoxic 58As9 cells. Further investigation may solve this question.

We next demonstrated that additional GI to 10 μM YC-1 induced hypoxia-dependent apoptosis more strongly than YC-1 mono-treatment. Low-dose YC-1 or YC-1 + GI exhibited similar effects in another GC cell line, MKN74, indicating hypoxia-dependent cell death by these treatments was not restricted to one cell line. A previous study reported that 100 μM YC-1 inhibited HIF-1α expression and induced apoptosis in PC3 cells under hypoxia^29^. However, this study did not evaluate YC-1 under normoxia. Another study reported that 1 μM YC-1 inhibited Hep3B proliferation under both normoxia and hypoxia^30^. Therefore, we showed for the first time that hypoxia-dependent apoptosis is induced by 10 μM YC-1 in GC cells.

Thereafter, we showed YC-1 or YC-1 + GI time-dependently accumulated ROS in hypoxic 58As9 cells, and higher ROS was generated in YC-1 + GI than YC-1. NAC reversed cell death by YC-1 + GI in hypoxic cells, indicating apoptosis was induced by excessive ROS generation. RT-qPCR analysis revealed hypoxic induction of the HIF-1α targets GLUT1, ALDOC, PDK1 and MCT4 was attenuated by YC-1 and YC-1 + GI to similar degrees. These results implied HIF-1α inhibition by YC-1 suppressed anaerobic glycolysis via reducing expression of these genes. However, the inhibitory effect of YC-1 or YC-1 + GI on HIF-1α targets was weaker than hypoxic KD cells. Additionally, hypoxic LDHA induction was not affected by 10 μM YC-1 treatment. Higher doses of YC-1 than 10 μM may be necessary for stronger HIF-1α inhibition, thereby hypoxic induction of the HIF-1α targets GLUT1, ALDOC, LDHA, PDK1 and MCT4 may be more strongly suppressed.

We further investigated the biological effect of YC-1 + GI on glucose metabolism. To assess the effect of YC-1 or YC-1 + GI on glucose uptake, the 2DG uptake test was performed with or without YC-1 ± I. 2DG uptake was strongly accelerated by hypoxic stimuli in control cells, which was further elevated by insulin. The promotion of 2DG uptake by hypoxia was smaller in YC-1 (10 μM) compared with controls. WB analysis showed that membranous GLUT1 expression was elevated by GI treatment in control cells under hypoxia, while it was entirely reduced by YC-1 under hypoxia; however, membranous GLUT1 was increased by GI treatment. This suggested that the strong elevation of glucose uptake in hypoxic control cells was due to GLUT1 up-regulation via HIF-1α activation, and the uptake was further promoted by insulin, which enhanced GLUT1 membrane translocation. Insulin signalling may stimulate GLUT1 translocation from intracellular storage vesicles to the plasma membrane as was reported for GLUT4 in adipocytes^31^. Conversely, the hypoxic stimulation of glucose uptake was smaller in 10 μM YC-1 than controls. RT-qPCR showed that the hypoxic induction of GLUT1 mRNA was attenuated by YC-1, which may lead to weaker stimulation of glucose uptake by hypoxia in YC-1-treated cells. However, additional GI sustained increased GLUT1 translocation, and may contribute to promotion of glucose uptake.

Metabolic analysis showed that the OCR/ECAR ratio was significantly elevated by YC-1 in 58As9 cells under hypoxia-mimetic conditions. These results suggested that YC-1 switched glucose metabolism from anaerobic glycolysis to OXPHOS in hypoxic cells. Assessments of glucose metabolites further revealed that 10 μM YC-1 elevated intracellular acetyl-CoA in hypoxic 58As9 cells, while the treatment decreased extracellular lactate. These results suggested that reduced PDK1 expression allows the conversion of pyruvate to acetyl-CoA but not lactate in YC-1-treated cells under hypoxia, which results in the elevation of intracellular acetyl-CoA instead of lactate; furthermore reduced MCT4 expression may decrease lactate efflux. This metabolic reprograming, derived from HIF-1α inhibition by YC-1, may generate excessive ROS and induce hypoxia-dependent apoptosis in YC-1-treated cells. Moreover, additional GI may elevate glucose uptake through membranous GLUT1 translocation in YC-1 treated cells under hypoxia. Thereafter, larger amounts of acetyl-CoA may be produced through glycolysis, inducing further ROS production in the OXPHOS pathway, causing more apoptosis than YC-1 mono-treatment.

Finally, we analyzed the *in vivo* effect of YC-1 + GI treatment using a tumour xenograft model. YC-1 is known to prevent intravascular thrombus formation by inhibiting platelet aggregation^27^. A previous study reported that YC-1 ip injections (10, 30 mg/kg) prolonged tail bleeding-time in mice^27,28^. Hence, we determined 1 mg/kg of YC-1 as the optimal dose for preclinical study. Doses of GI at glucose (2 g/kg) and insulin (1 unit/3 g glucose) were determined, because higher doses of glucose (4 or 8 g/kg) resulted in serious hyperglycemia (supplement Fig. 3). The results demonstrated that YC-1 + GI strongly suppressed xenograft tumour growth, while YC-1 mono-treatment incompletely blocked growth. WB analysis showed HIF-1α expression in control 58As9 tumours, suggesting hypoxic regions persisted. In contrast, HIF-1α expression was inhibited in YC-1- or YC-1 + GI-treated tumours, but apoptosis markers were more strongly induced by YC-1 + GI than YC-1. Immunohistochemistry findings indicated that YC-1 + GI inhibited HIF-1α expression in xenograft tumours, and this combination coincidently decreased pimonidazole expression. Conversely, YC-1 + GI increased cleaved-caspase3 and 8-OHdG levels in tumours. Thus, YC-1 + GI treatment selectively inhibited hypoxic cancer cell growth in xenograft tumours. In these hypoxic cells, YC-1 inhibits anaerobic glycolysis via HIF-1α suppression, and additional GI promotes glucose uptake. These dual effects of YC-1 + GI synergistically elevate acetyl-CoA through glycolysis, and induce lethal ROS production.

**Figure 8:**
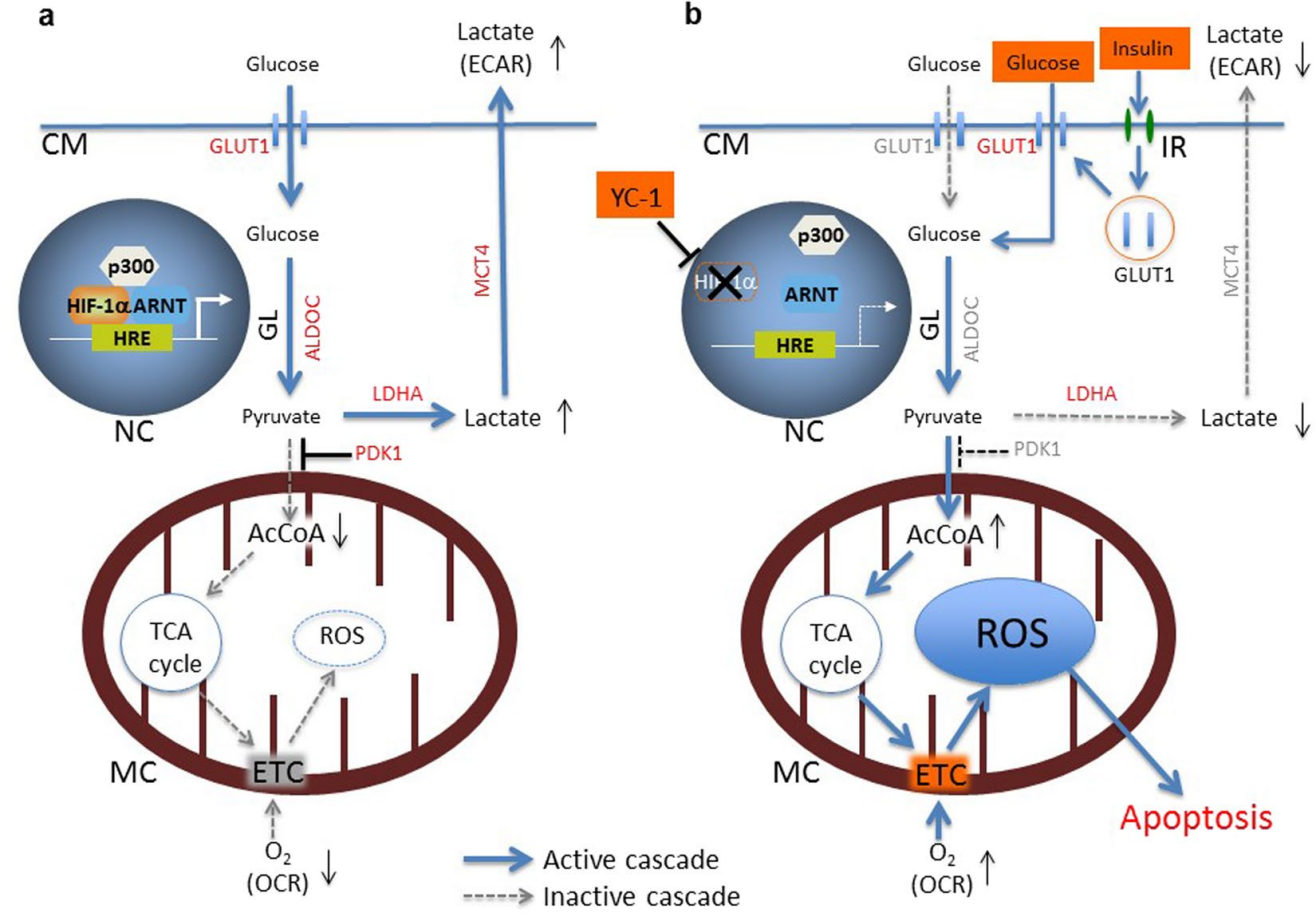
*In vitro* apoptotic effect of low-dose YC-1 + GI treatment on hypoxic 58As9 cells. (**a**) HIF-1α controls ROS production in hypoxic 58As9 cells by up-regulating genes involved in anaerobic glycolysis such as GLUT1, ALDOC, LDHA, PDK1 and MCT4 (indicated by red letters). (**b**) Low-dose YC-1 + GI treatment induces hypoxia-dependent apoptosis in 58As9 cells. YC-1 treatment inhibits HIF-1α expression in hypoxic 58As9 cells and suppresses up-regulation of the HIF-1α targets GLUT1, ALDOC, PDK1 and MCT4 (indicated by gray letters). Attenuation of the expression of these genes causes a metabolic switch from anaerobic glycolysis to OXPHOS in hypoxic 58As9 cells, resulting in the elevated OCR/ECAR ratio. Additionally, GI treatment accelerates GLUT1 membrane translocation (indicated by red letters) via insulin signalling, promotes glucose uptake in hypoxic 58As9 cells, and contributes to increased AcCoA entry into the TCA cycle. These dual effects of low-dose YC-1 + GI treatment result in lethal ROS production through the ETC. CM: cell membrane, NC: nucleus, HRE: hypoxia responsive element, MC: mitochondria, GL: glycolysis, IR: insulin receptor. AcCoA: acetyl-CoA, OCR: oxygen consumption rate, ECAR: extracellular acidification rate.

In summary, *in vitro* apoptotic mechanism of low-dose YC-1 + GI treatment in hypoxic 58As9 cells is illustrated in Fig. 8. This study revealed that low-dose YC-1 + GI therapy targets hypoxic cancer cells, where a metabolic switch from anaerobic glycolysis to OXPHOS is induced under hypoxia, resulting in ROS-mediated apoptosis. Additionally, this treatment may supply glucose to normal cells that live under normoxic environments. Therefore, low-dose YC-1 + GI may be an attractive anti-cancer therapy because hypoxic cancer cells with malignant behaviors may be selectively killed, while normal cells will survive and receive energy from the GI treatment.

## Methods

### Cell culture conditions and reagents

The GC cell line 58As9 was established and kindly provided by Dr. K. Yanagihara (National Cancer Center, Chiba, Japan)^32^. This cells line was further authenticated by the JCRB Cell Bank (Osaka, Japan). MKN74 cells were purchase from Cell Bank, RIKEN Bio Resource Center (Tsukuba, Japan). HIF-1α knockdown (KD) cells were established by transfecting plasmids harboring HIF-1α RNAi sequences into 58As9 and MKN74 cells, as previously described^11,16^. The cells were cultured at 37 °C in RPMI-1640 medium (Sigma-Aldrich, St. Louis, MO, USA) supplemented with 10% heat-inactivated fetal bovine serum and 100 μg/mL kanamycin (Meiji, Tokyo, Japan). Cells were cultured under either normoxic (20% O_2_ and 5% CO_2_ in air) or hypoxic conditions (1% O_2_, 5% CO_2_ and 94% N_2_) in a hypoxic chamber (ASTEC, Fukuoka, Japan). YC-1 (Sigma-Aldrich), NAC (Sigma-Aldrich) and Insulin (Wako, Osaka, Japan) were used at a final concentration of 10 µM, 5 mM and 500 ng/ml, respectively. High glucose medium was prepared using a 45% D-(+)-Glucose solution (Sigma-Aldrich) and the concentration was determined to be 10 g/L, 5 times higher than RPMI-1640 medium.

### Cell viability assay

Cell viability was assessed by the MTS assay using the CellTiter 96®AQueous One Solution Cell Proliferation Assay kit (Promega, Madison, WI, USA), as previously described^11^. The cell death rate was estimated by trypan blue dye exclusion method, as previously described^16^. These experiments were independently repeated at least three times.

### Western blot (WB) analysis

Whole cell lysates from cultured cells and mouse tumors were prepared as previously described^16^. Cell lysates from the cell membrane fraction were prepared using Plasma Membrane Protein Extraction Kit (BioVision Inc., Milpitas, CA, USA) according to the manufacturer’s instructions. WB analysis was performed as previously described^16^. using the following primary antibodies: anti-HIF-1α (1:1000, clone54/HIF1α, BD-Biosciences, Franklin Lakes, NJ, USA), anti-cleaved caspase3 (1:1000, Cell Signaling Technology, Danvers, MA, USA), anti-cleaved PARP (1:1000, Cell Signaling Technology), anti-GLUT1 (1:1000, Abcam, Cambridge, UK) and anti–β-actin (1:10,000, Sigma-Aldrich).

### Detecting intracellular ROS by flow cytometry

Intracellular ROS levels were evaluated using the Total ROS Detection Kit (Enzo Life Sciences, Inc., Farmingdale, NY, USA) according to the manufacturer’s instructions as previously described^16^. ROS fluorescence was detected using a FACSCalibur flow cytometer (Becton-Dickinson, San Jose, CA, USA) and analyzed by the Cell Quest program to determine mean fluorescence.

### Total RNA extraction and real-time qPCR (RT-q**PCR**)

Total RNA extraction, followed by cDNA conversion was done as previously described^11^. RT-qPCR was performed using the Light Cycler instrument system (Roche Diagnostics GmbH, Mannheim, Germany) as previously described^16^. Five genes were analyzed by RT-qPCR: glucose transporter 1 (GLUT1), aldolase C (ALDOC), pyruvate dehydrogenase kinase 1 (PDK1), lactate dehydrogenase A (LDHA) and monocarboxylate transporter 4 (MCT4). Primers were designed according to the reported cDNA sequences (GenBank, Bethesda, MD, USA), and the sequences are shown in previous study^16^. All experiments were performed in triplicate, and mean values were calculated.

### Glucose uptake assay

Glucose uptake in cultured cells was determined using a 2-Deoxyglucose (2-DG) Uptake Measurement Kit (COSMO BIO Co. Ltd., Tokyo, Japan) as previously described^16^. Briefly, cells were cultured under a serum-starved condition for 6 h, followed by further culture for 18 h in regular medium supplemented with 10% FBS. The cells were then incubated for 24 h under normoxia or hypoxia. Thereafter, the cells were treated with or without 500 ng/ml insulin for 18 h. Finally, the cells were treated with 2DG for 20 min and subjected to 2DG uptake measurements according to the manufacturer’s instruction. All experiments were performed in triplicate, and mean values were calculated.

### Oxygen consumption rate (OCR) and extracellular acidification rate (ECAR) measurement

The mitochondrial OCR and ECAR were measured using Seahorse XFp Extracellular Flux Analyzer (Seahorse Bioscience, North Billerica, MA) as described previously^33^. Briefly, 3 × 104 cells were seeded in each well of Seahorse XFp Cell Culture Miniplates coated with poly-L-lysine solution (Sigma-Aldrich, St. Louis, MO) two days prior to the assay. The cells were exposed to cobalt chloride (CoCl2) (Sigma-Aldrich) at 100 μM final concentration for 16hr to create the mimic hypoxia. Subsequently, the cells were treated with or without YC-1 (10 μM) for 24 h. Before performing the glycolysis stress test, the culture medium was removed from each well, and then the cells were washed 2 times and filled by the assay medium for Seahorse adjusted the pH to 7.4 ( ± 0.02). Thereafter, the glycolysis stress test was employed by sequential injections of glucose (10 mM), oligomycin (2.5 μM) and 2-deoxy-glucose (2-DG) (50 mM). OCR and ECAR under basal and glucose-stimulated conditions were evaluated as means of values at the three time points before and after the addition of glucose, respectively.

### Evaluation of acetyl-CoA and lactate levels

Intracellular acetyl-CoA was measured using a Pico Probe™ Acetyl-CoA Fluorometric Assay Kit (BioVision Inc.). Extracellular lactate was measured using a Lactate Colorimetric/Fluorometric Assay Kit (BioVision Inc.).

### Animal experiments

All methods were performed in accordance with the relevant guidelines and regulations. Further, all animal protocols were approved by the Animal Care Committee of Saga University. Female athymic BALB/cA Jcl mice (nu/nu, 4-weeks-old) were obtained from Nihon Crea Co. (Osaka, Japan), kept under specific-pathogen-free conditions and given sterile food and autoclaved water. To establish the tumour models, 3 × 10^6^ 58As9 cells were subcutaneously injected into the back of the mice. One week after inoculation, xenografts became palpable. The 20 xenograft-bearing mice were divided into four treatment groups as follows: Control [phosphate-buffered saline (PBS)], GI (glucose: 4 g/kg/day, insulin: 1 unit per 3 g-glucose/day), YC-1 (1 mg/kg) and YC-1 + GI (above treatments combined). All drugs were intraperitoneally (ip) administered every 24 h from day 1 to day 14. During treatment, tumours sizes were measured along 2 perpendicular dimensions with a caliper every 4 d. Tumour size (*T*) was evaluated as the maximum cut area and determined by the formula: *T* = π/4 × *a* × *b*, where *a* (mm) is the shorter axis and *b* (mm) is the longer axis.

### Immunohistochemistry

After deparaffinization and rehydration, antigen retrieval was performed with high pH CC1 buffer at 99 °C for 1 h (Ventana Medical Systems, Inc., Tucson, AZ, USA). For immunostaining, 4-μm sections were incubated with antibodies against HIF-1α (1:50), pimonidazole (1:100, clone Pab2627, rabbit polyclonal, COSMO BIO Co. Ltd., Tokyo, Japan), cleaved caspase-3 (1:50) and 8-hydroxy-2´-deoxyguanosine (8-OHdG) (1:50, N45.1, mouse monoclonal, Japan Institute for Control of Aging, Fukuroi, Japan). Slides were incubated with primary antibodies at 4 °C overnight, and immunohistochemical staining was performed with the EnVision + system (Dako, Glostrup, Denmark). The sections were then treated with 3,30-diaminobenzidine and counterstained with hematoxylin.

### Evaluation of immunohistochemical staining

The proportion of positively-stained nuclei for HIF-1α and 8-OHdG, or cytoplasm for pimonidazole and cleaved caspase-3 were assessed in the central region of tumours, and semi-quantitatively scored by a pathologist. The proportion of stained cells was evaluated in three fields of hot-spot areas at high power (200× ) and scored from 0–100%.

### Statistical Analysis

Data were analyzed by ANOVA using Prism 5 software (GraphPad Software, La Jolla, CA, USA). For comparisons between two groups, the differences in mean values were evaluated by Student’s *t-*test and Mann–Whitney U test. For comparisons among three or more groups, Bonferroni post-hoc tests were performed for One-way ANOVA. A value of p < 0.05 was considered statistically significant. All values are expressed as means ± SEM.

## Electronic supplementary material

Supplemental figure 1

Supplemental figure 2

Supplemental figure 3
