# Supplementary figures and images for: Low-dose YC-1 combined with glucose and insulin selectively induces apoptosis in hypoxic gastric carcinoma cells by inhibiting anaerobic glycolysis
Source: Sci Rep. 2017 Oct 4;7:12653. doi: 10.1038/s41598-017-12929-9 (PMC5627264; doi:10.1038/s41598-017-12929-9)

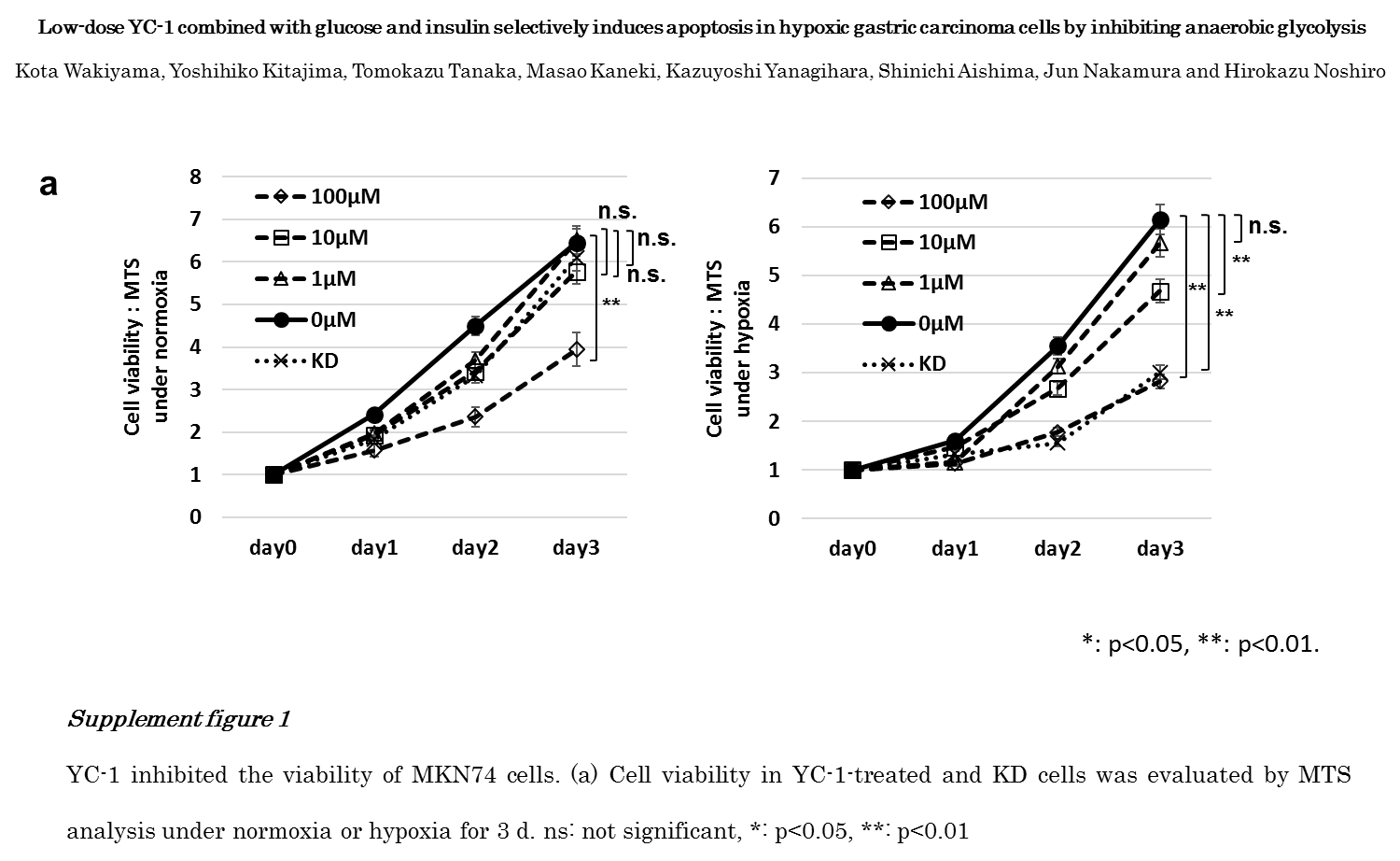

Supplement: Supplementary file 1 — Supplemental figure 1 [file 41598_2017_12929_MOESM1_ESM.doc]

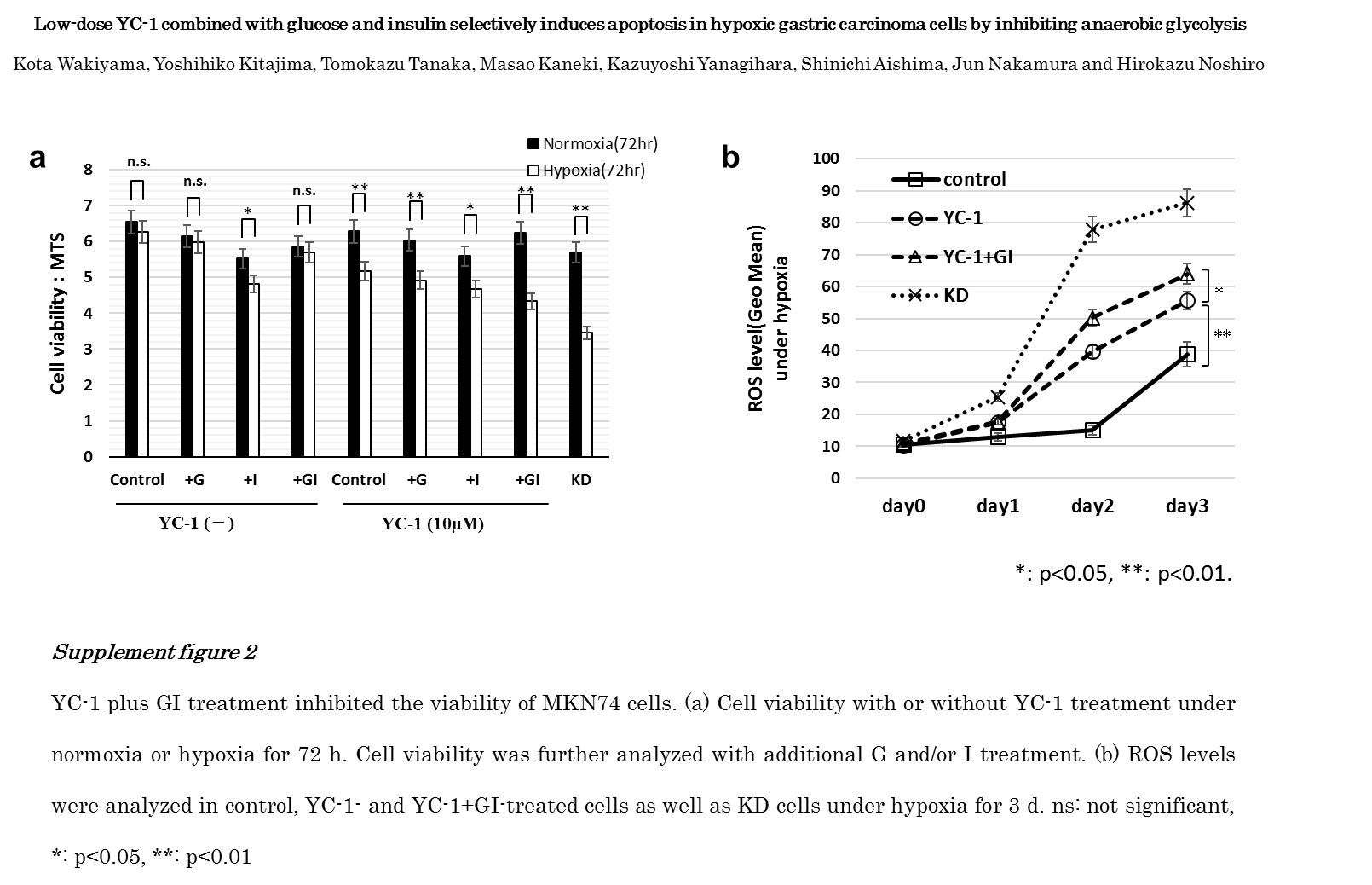

Supplement: Supplementary file 2 — Supplemental figure 2 [file 41598_2017_12929_MOESM2_ESM.doc]
