# Supplementary material for: Low-dose YC-1 combined with glucose and insulin selectively induces apoptosis in hypoxic gastric carcinoma cells by inhibiting anaerobic glycolysis
Source: Sci Rep. 2017 Oct 4;7:12653. doi: 10.1038/s41598-017-12929-9 (PMC5627264; doi:10.1038/s41598-017-12929-9)

**Low-dose YC-1 combined with glucose and insulin selectively induces apoptosis in hypoxic gastric carcinoma cells by inhibiting anaerobic glycolysis**

Kota Wakiyama, Yoshihiko Kitajima, Tomokazu Tanaka, Masao Kaneki, Kazuyoshi Yanagihara, Shinichi Aishima, Jun Nakamura and Hirokazu Noshiro


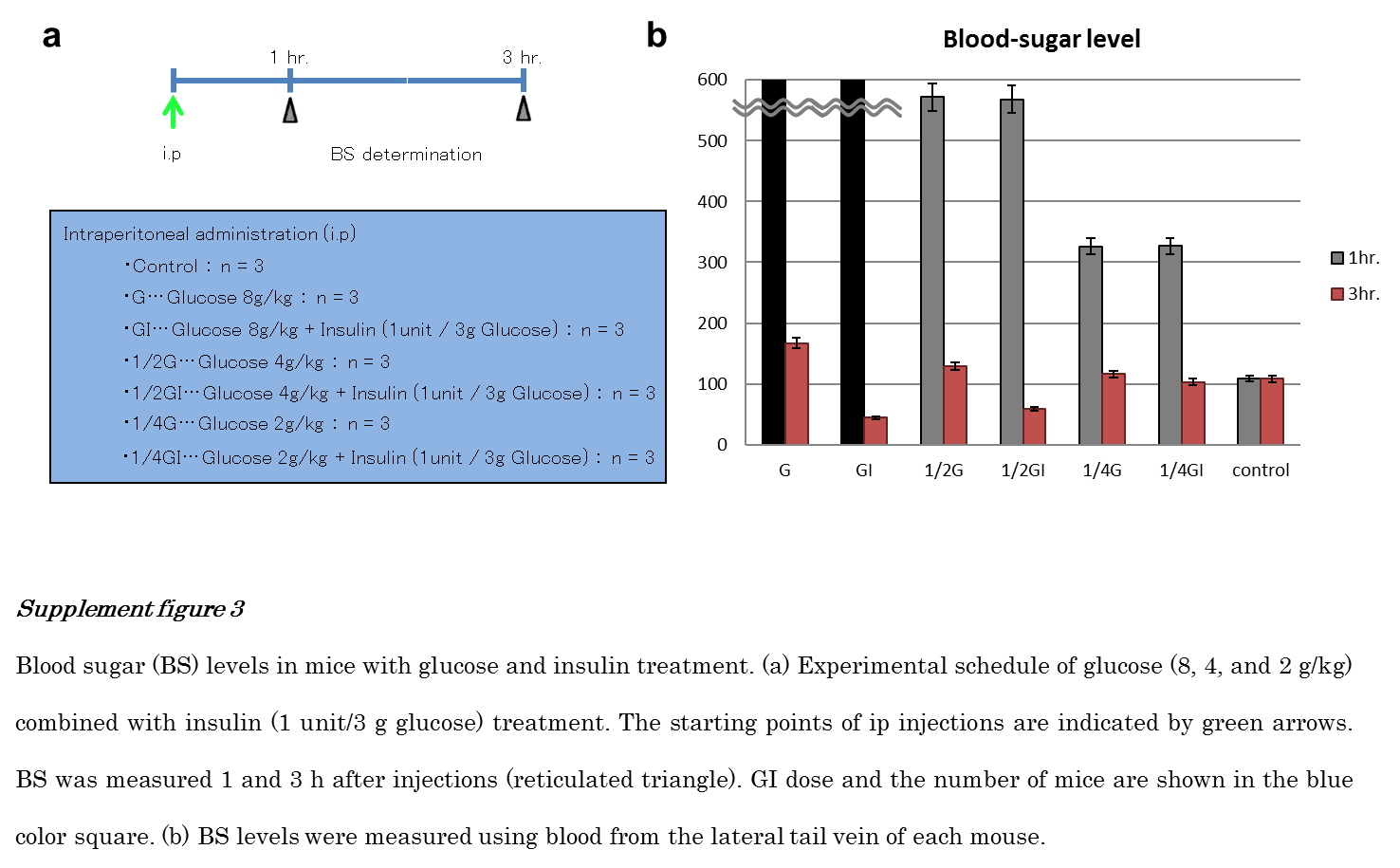

Supplement: Supplementary file 3 — Supplemental figure 3 [file 41598_2017_12929_MOESM3_ESM.doc]
